# A scoping review of factors influencing the implementation of liquid biopsy for cancer care

**DOI:** 10.1186/s13046-025-03322-w

**Published:** 2025-02-12

**Authors:** Samran Sheriff, Maree Saba, Romika Patel, Georgia Fisher, Tanja Schroeder, Gaston Arnolda, Dan Luo, Lydia Warburton, Elin Gray, Georgina Long, Jeffrey Braithwaite, Helen Rizos, Louise Ann Ellis

**Affiliations:** 1 Centre for Healthcare Resilience and Implementation Science, Australian Institute of Health Innovation, Macquarie University, Level 6, 75 Talavera Road, North Ryde, Sydney, NSW Australia; 2 The Daffodil Centre, Sydney, NSW Australia; 3 Centre for Precision Health, Edith Cowan University, Joondalup, WA Australia; 4 Department of Medical Oncology, Fiona Stanly Hospital, Murdoch, WA Australia; 5 School of Medical and Health Sciences, Edith Cowan University, Joondalup, WA Australia; 6 Melanoma Institute Australia, The University of Sydney, Sydney, NSW Australia; 7 Faculty of Medicine & Health, The University of Sydney, Sydney, NSW Australia; 8 Charles Perkins Centre, The University of Sydney, Sydney, NSW Australia; 9 Royal North Shore and Mater Hospitals, North Sydney, Sydney, NSW Australia; 10 Macquarie Medical School, Faculty of Medicine Health and Human Science, Macquarie University, Sydney, NSW Australia

**Keywords:** Liquid biopsy, Cancer care, Barriers, Facilitators, Implementation, Personalised medicine, CtDNA, CfDNA, CTC

## Abstract

**Background:**

Liquid biopsy (LB) offers a promising, minimally invasive alternative to traditional tissue biopsies in cancer care, enabling real-time monitoring and personalized treatment. Despite its potential, the routine implementation of LB in clinical practice faces significant challenges. This scoping review examines the barriers and facilitators influencing the implementation of liquid biopsies into standard cancer care.

**Methods:**

Four academic databases (PubMed, Scopus, Embase, and Web of Science) were systematically searched without language restrictions. We included peer-reviewed articles that were published between January 2019 and March 2024 that focused on the implementation of LB in cancer care or described barriers and facilitators to its implementation. Data relevant to the review objective, including key article characteristics; barriers and facilitators of implementation; and recommendations for advancement or optimisation; were extracted and analysed using thematic and visual network analyses.

**Results:**

The majority of the included articles were narrative review articles (84%), with most from China (24.2%) and the United States (20%). Thematic analysis identified four main categories and their associated barriers and facilitators to the implementation of LB in cancer care: (1) Laboratory and personnel requirements; (2) Disease specificity; (3) Biomarker-based liquid biopsy; and (4) Policy and regulation. The majority of barriers identified were concentrated in the pre-analytical phase, highlighting the lack of standardization in LB technologies and outcomes.

**Conclusions:**

Through a thematic analysis of the barriers and facilitators to LB implementation, we present an integrated tool designed to encourage the standardization of testing methods for clinical practice guidelines in the field.

**Supplementary Information:**

The online version contains supplementary material available at 10.1186/s13046-025-03322-w.

## Introduction

Cancer remains the leading cause of morbidity and mortality worldwide, with an estimated 20 million new cases and 9.7 million cancer-related deaths in 2022 [1]. Given this burden, there is increasing demand for personalised cancer management and treatment selection to improve survival rates and enhance patient care. Population screening methods are available as secondary prevention strategies for some cancers but rely on invasive and expensive procedures such as bowel screening, mammograms and Pap smears [2]. These screening programs face challenges such as low predictive accuracy, leading to false positives, unnecessary interventions, and missed diagnoses. Non-screening diagnosis usually commences with imaging or blood-based assays to investigate the causes underlying presenting signs and symptoms. In either case, formal diagnosis typically requires tissue biopsy for histological confirmation of cancer type to guide initial treatment, and mutational profiling can also inform therapy selection [3, 4]. The development of sensitive and specific non-invasive techniques to complement traditional biopsy methods are evolving and have the potential to enhance cancer diagnosis and improve patient outcomes [5, 6].

Over the past 20 years, advancements in precision medicine and innovative biomedical technologies have revolutionised cancer care, enabling more individualised treatment plans based on patient-specific biomarkers [7–9]. However, significant challenges remain in fully realising the potential of personalised treatments, including tissue inaccessibility, intra-tumour heterogeneity (which may be missed by tissue biopsy), inter-tumour heterogeneity (metastases may have distinct molecular profiles), and the expense and time required for repeated sampling [10–15]. Minimally invasive techniques, offering real-time analysis of predictive and prognostic biomarkers, could help address these challenges, especially where tissue biopsy is difficult (e.g., lung or brain cancers) [16].

**Fig. 1:**
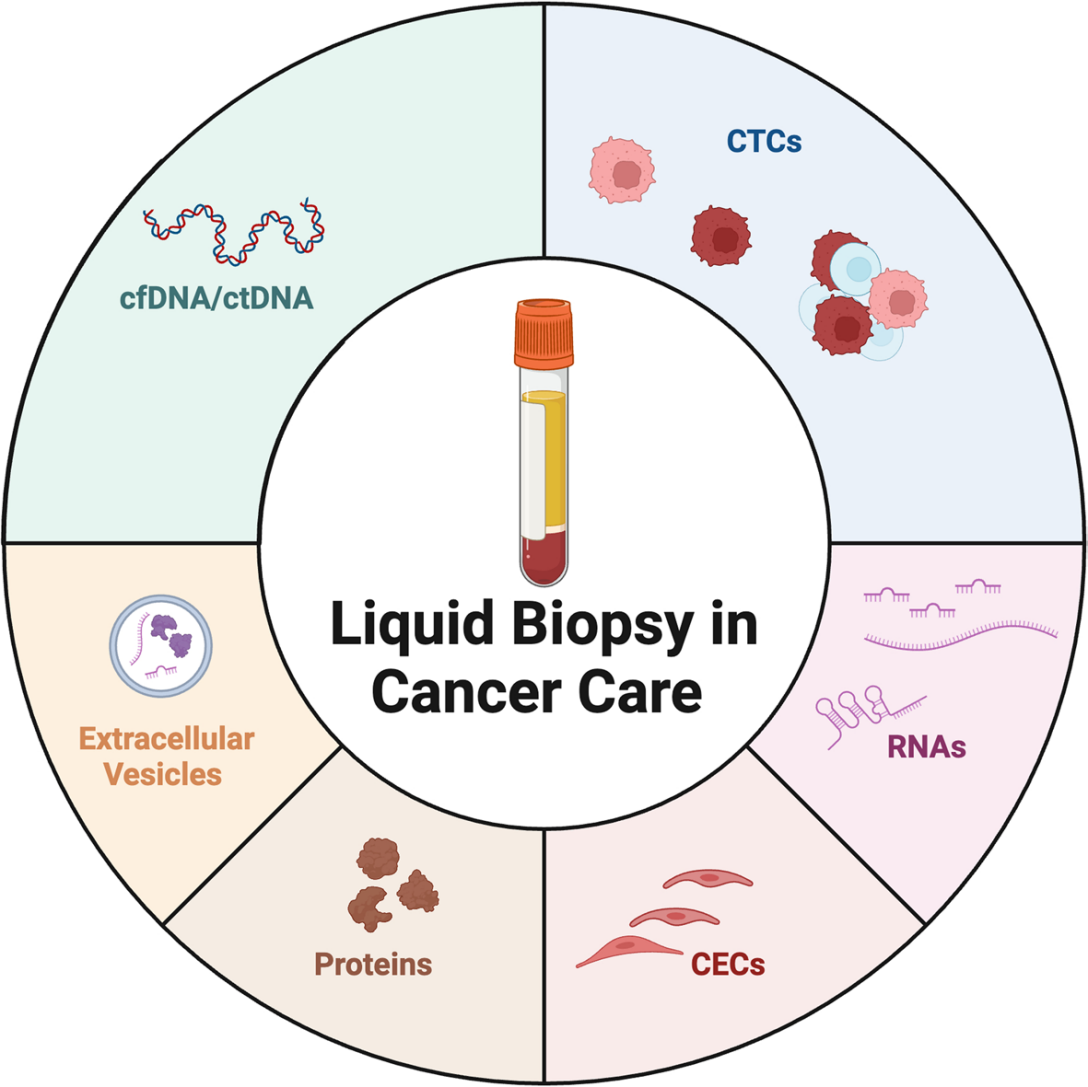
Common analytes for Liquid Biopsy. Liquid biopsies are typically obtained via blood sampling, but can also be derived from urine, cerebrospinal fluid (CSF), ascites fluid, and pleural fluid. These biopsies contain several cancer related biomarkers including cell free DNA/circulating tumour (cfDNA/ctDNA), Circulating tumour cells (CTCs), circulating cell-free RNA (cfRNA), and extracellular vesicles. Exosomes, CEC (circulating endothelial cells) and proteins are biomarkers present in the blood that also serve as common targets for LB

Liquid biopsy (LB) is a minimally invasive approach that utilises fluid samples such as urine, blood, saliva and cerebrospinal fluid, to detect tumour cells or biomarkers reflecting tumour cell activity and burden [17, 18]. Biomarkers commonly explored in LB include circulating tumour cells (CTC); circulating tumour DNA (ctDNA) which is a subset of cell-free DNA (cfDNA); extracellular vesicles; proteins; and circulating RNA species (Fig. 1). CTCs are complete tumour cells that have entered the bloodstream [19, 20], while ctDNA consists of small fragments released by tumour cells, that may be undergoing apoptosis or necrosis or may be actively secreted [21, 22]. Tumour-derived extracellular vesicles are small vesicles produced by tumour cells to transport nucleic acids and proteins that reflect tumour cellular processes and the tumour microenvironment [23, 24]. Cell-free RNA (cfRNA) is also used as a biomarker in cancer, with most research focussed on stable non-coding RNA species including long noncoding RNAs, microRNAs and circular RNAs [25, 26]. Circulating endothelial cells are endothelial cells that have been shed from the lining of the vascular wall into the blood stream indicating possible vascular dysfunction and damage [27].

**Fig. 2:**
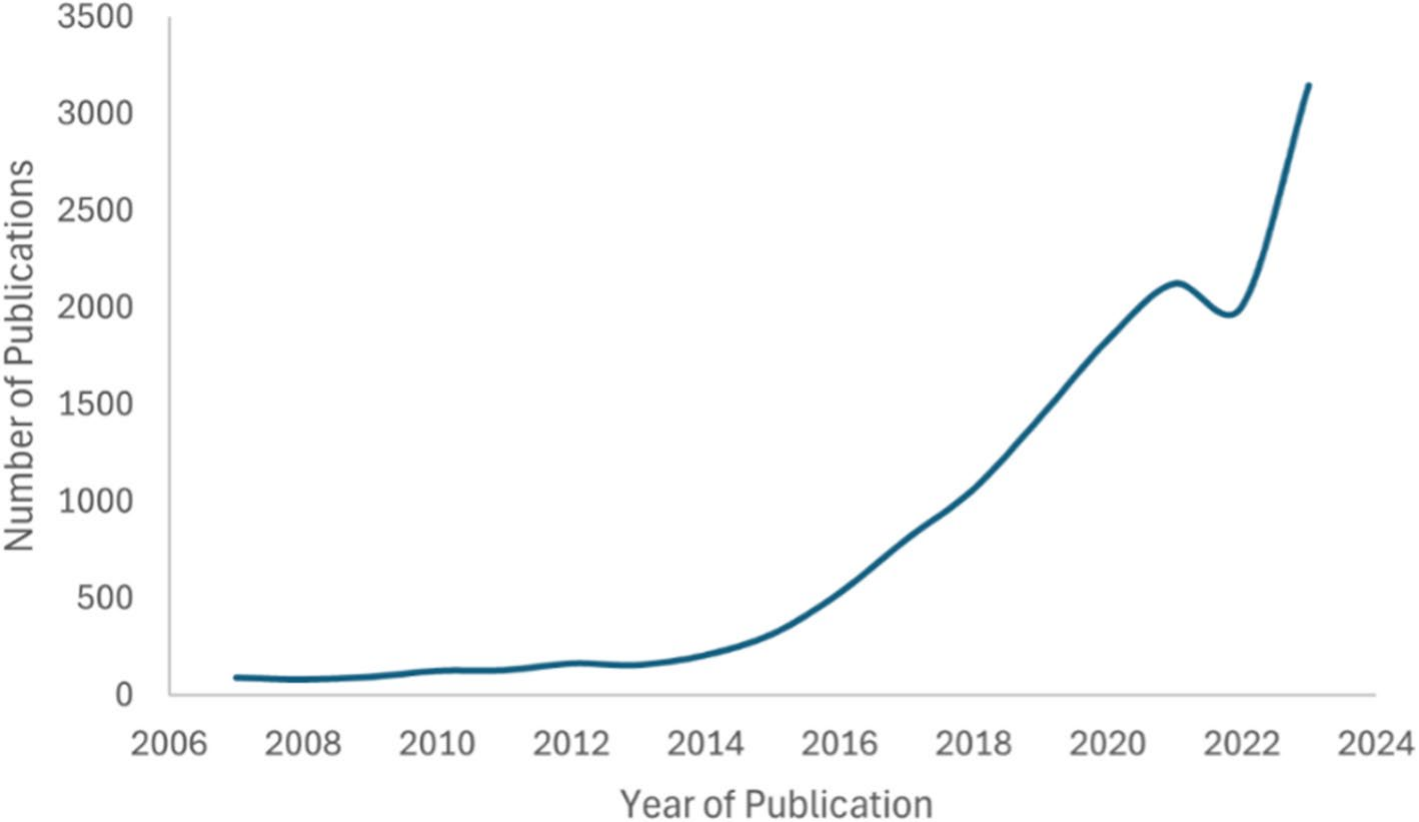
Annual number of Liquid Biopsy publications between 2007 and 2023. Publication numbers were derived from Medline featuring “Liquid Biopsy” in the title or abstract

LB biomarkers can be used for the earlier detection of cancers, improve information available for initial treatment selection and support real-time monitoring of treatment efficacy and tumour progression [7, 11, 15, 28–30]. Despite extensive interest, LB is yet to be widely adopted in clinical practice [15, 31]. Growing interest in LB is evidenced by the sharp increase in the number of publications on ‘liquid biopsy’ since 2014 (Fig. 2).

LB has the potential to transform clinical practice by offering a less invasive, real-time approach to the identification of specific biomarkers, enabling early detection and monitoring [32]. The clinical utility of LB (particularly ctDNA) in various cancer types is increasing, especially for the purpose of minimal residual disease (MRD) detection. Phase III studies have been and are being undertaken, such as the DYNAMIC II and III trial and COBRA trial in colorectal cancer [33, 34]; AURA3, TRACERx and MERMAID-2 in NSCLC (non-small cell lung cancer) [35–37]; ZEST in Breast cancer and IMvigor011 in bladder cancer [38, 39]. These studies highlight the potential of LB to transform cancer care, providing a more personalised and less invasive method for detecting disease and monitoring treatment responses. However, significant barriers persist, including the variability in assay sensitivity and specificity, the lack of standardization across different LB platforms, and the limited research focused predominantly on specific cancer types rather than a comprehensive, systematic exploration of its broader applications [40–42]. Furthermore, many studies reported in the literature are non-systematic reviews and lack an implementation science approach, which may not provide a complete picture of the LB capabilities and limitations.

### The current study

With the growth of interest and research on LB, it is timely to examine the published literature to identify the factors that support or inhibit the successful implementation of LB in cancer care. With an abundance of literature available and a lack of clear implementation strategies, we conducted a scoping review to identify and categorise these factors and propose practical next steps for expediting the integration of LB into standard cancer care practices.

The specific objectives of this scoping review were to:

1. Identify published literature pertaining to LB use in cancer care.
2. Synthesise this literature and identify the key factors that influence the implementation of LB in cancer care.
3. Provide recommendations for the implementation of and future directions for LB in cancer care.

## Methods

This review followed a prespecified protocol, developed in accordance with the Preferred Reporting Items for Systematic Reviews and Meta-Analyses extension for Scoping Reviews (PRISMA-ScR) guidelines and the Joanna Briggs Institute Methodology for Scoping Reviews [7, 11, 15, 43]. The review protocol was pre-registered on Open Science Framework: https://osf.io/nqaj4. A scoping review methodology was employed to examine the extent, range, and nature of work on this topic. The review focus was to identify gaps and provide recommendations to improve future directions for research and practice on the barriers and enablers associated with the implementation of LB in cancer care. Quality assessments were not conducted, as the aim was to examine the full breadth of the literature, consistent with the general aims and methodology of scoping reviews [44].

### Search strategy

The initial search was performed by two independent Reviewers (SS and MS) using medical subject headings (MeSH) terms and subject headings. Four academic databases (PubMed, Scopus, Embase, and Web of Science) were systematically examined without language and date restrictions up to the end of March 2024. Medical subject headings and keywords identified from relevant literature that were related to LB, cancer, and clinical use were applied. Search terms used encompassed keyword concepts relating to: (1) LB (e.g., circulating tumour, exosome, cell-free nucleic acid, ctDNA, cfDNA); (2) implementation factors (e.g., barrier, challenge); (3) cancer (e.g., oncology, carcinoma, tumour); and (4) context (e.g., clinic, hospital, trial). In addition to database searching using MeSH terms and subject headings, we also manually reviewed and searched the reference lists of initially selected articles to avoid missing relevant studies. Articles were required to fulfill the criteria for both concepts 1 and 2, and at least one of concepts 3 or 4. The search strategy was developed in consultation with the research team and was reviewed by all authors prior to execution. The specific search criteria used in this review are detailed in Supplementary File 1. 

### Inclusion and exclusion criteria

Publications were included if they: (1) explicitly mentioned at least one barrier or enabler to the implementation of LB in cancer clinical practice; (2) referred to LB or associated biomarkers (specifically one or more of CTC, ctDNA, cfDNA) used for cancer detection; and (3) were peer-reviewed articles published in English between 2019–2024. Publications were excluded if they: (1) solely evaluated the validity and reliability of LB procedures without mention of any other implementation factor; (2) solely described animal studies; or (3) were book chapters, protocols, or conference proceedings.

### Citation screening

Article details, including abstracts, were downloaded and imported into EndNote 20, then exported into the systematic review platform Rayyan QCRI [45]. Duplicates identified by Rayyan were manually reviewed and removed by a single author (MS). The study selection process involved two stages: title/abstract screening and full-text assessment. Members of the research team (SS, MS, RP, GA, LAE, TS) independently screened the title/abstracts for eligibility against the criteria, with 5% of titles/abstracts being blind screened by the entire review team to ensure consistent application of the criteria; each of the six primary reviewers was assigned an equal share of these articles and agreement was assessed as majority agreement with the primary reviewers decision to include or exclude the title for full text screening (i.e., 3 or more of the other reviewers agreed). To ensure consistency and accuracy in the screening process, the primary reviewer’s screening results were systematically compared with the collective consensus of the review team, allowing for the resolution of any discrepancies through discussion. Included articles that met the criteria during the initial screening phase were then subjected to a thorough full-text review, conducted by three reviewers (SS, MS, RP). Throughout the review process, regular meetings were convened to discuss findings, address challenges in article selection, and ensure consistency of article inclusion.

### Data extraction

The elements for data extraction were determined through discussions among all authors. Data from included articles were extracted into a custom data extraction form developed in Microsoft Excel. This template was piloted by three members of the review team (SS, MS, RP) with a subset of 10 articles to evaluate the uniformity of data extraction and assess the form’s usability. Any issues with data entry consistency and template usability were then discussed and addressed accordingly. Following this, the remaining articles were distributed among five reviewers for full-text data extraction (SS, MS, RP, GA, TS).

Key information extracted included: article characteristics (i.e., authors, date of publication, country of the corresponding author, journal name); article keywords, as supplied by the authors of the paper; article type (i.e., empirical, nonempirical, review); tumour stream (e.g., colorectal, lung, head and neck); LB focus (i.e., ctDNA, cfDNA, CTC; barriers to the implementation of LB in clinical practice; facilitators to the implementation of LB in clinical practice; and strategies for advancing or optimising the implementation of LB.

### Data synthesis and analysis

Data were analysed by a single researcher (SS) using inductive qualitative content analysis [46]. Extracted data from articles were grouped into overarching factors that inhibit or facilitate the implementation of LB and analysed. The analysis was conducted in Microsoft Excel, starting with open coding, where as many ‘headings’ as necessary were inductively assigned to categorise all units of meaning within the extracted data. Following open coding, the categories were compared, refined, and consolidated into a coding framework. The country of the first author was coded by income classification based on World Bank definitions of the gross national income per capita. The three categories were low (< US $1135), middle (US $1136–$13,845), and high (> US $13,846) income [47].

Keywords were extracted and narratively synthesised into key topic areas. Derivative terms (e.g., Liquid Biopsy and LB and cfDNA and cfDNA) were amalgamated. Each keyword was reviewed and inductively classified by two authors (SS and LAE) into key topic. The keyword data were analysed for frequency and co-occurrence graphically presented using Gephi software, version 10.1. In these networks, the nodes (circles) represent the author generated keywords, while the ties (lines) indicate the co-occurrence of keywords within a single article. The size of each node reflects the frequency with which a keyword was identified [48].

## Results

### Overview

**Fig. 3:**
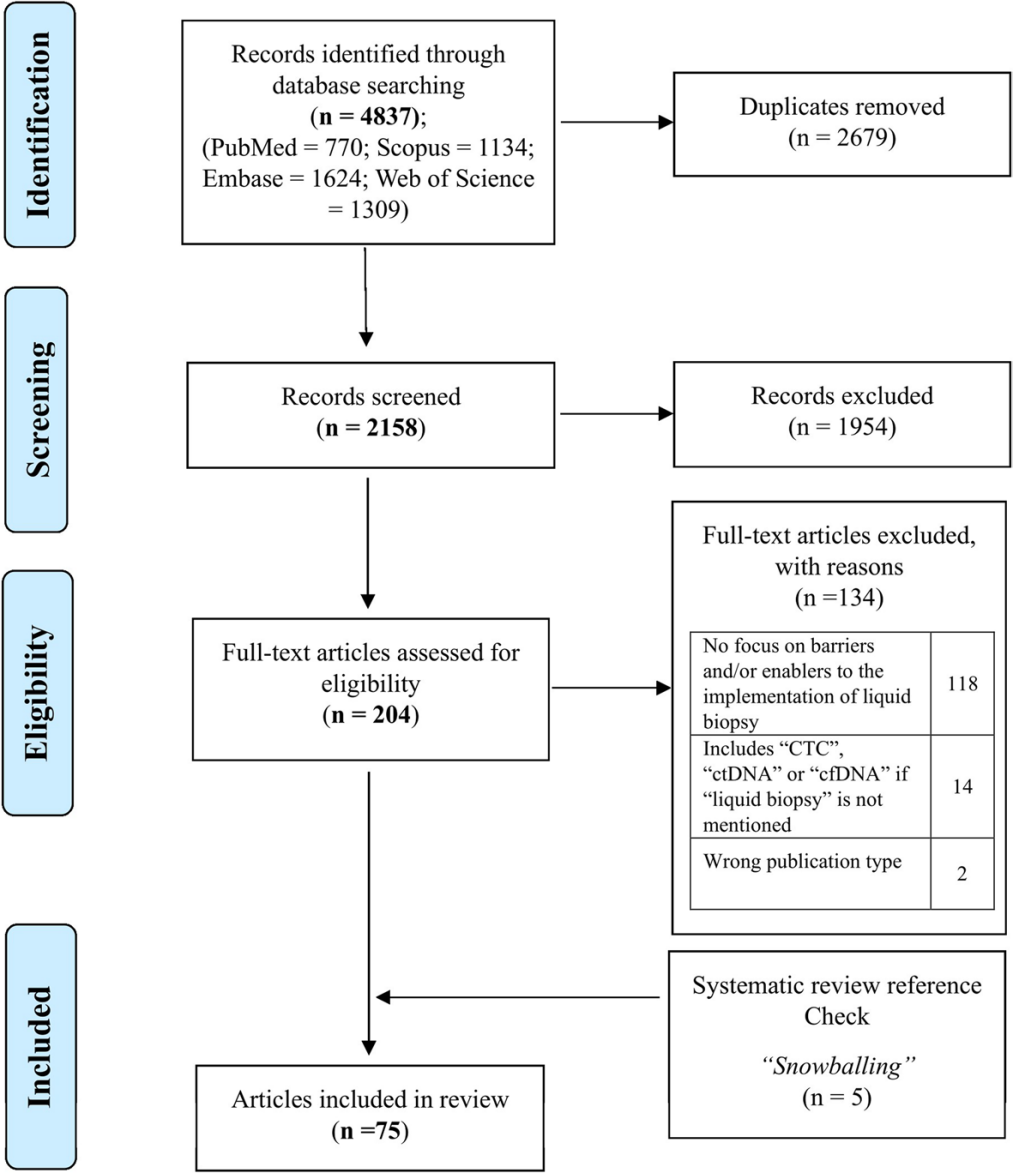
PRISMA flowchart displaying the process of identification and selection of included publications. Abbreviations: cell free DNA/circulating tumour (cfDNA/ctDNA), Circulating tumour cells (CTCs)

The search retrieved a total of 4,837 publications, of which 2,158 were unique records. Through title and abstract screening, 1,954 publications were excluded for not meeting the inclusion criteria (Fig. 3). The remaining 204 references underwent full-text screening and extraction, resulting in 70 publications being included in this review. Additionally, 5 publications were included from snowballing the reference lists of included publications. To validate the screening process, a total of 108 records (5%) of the 2158 unique records were screened, with the majority of the review team agreeing with the primary reviewer’s decision 90% of the time, demonstrating strong agreement (not shown in PRISMA). Figure 3 illustrates the inclusion and exclusion of records at each stage of the screening process.

### Publication characteristics

A summary of the key characteristics of the included articles is presented in Table 1. The corresponding and first authors were predominantly from high-income Organisation for Economic Co-operation and Development (OECD) countries, with the majority of these publications originating from the United States (*n* = 15, 20%), Australia (*n* = 5, 7.1%), Italy (*n* = 4, 5.7%), and Canada (*n* = 4, 5.7%). There was also a significant contribution from middle-income countries (*n* = 22, 31.4%), with China (*n* = 17, 24.2%) and India (*n* = 2, 2.8%) being the most commonly represented. The included studies primarily focused on lung (*n* = 8, 11.4%), colorectal (*n* = 7, 9.4%) and head and neck/oral (*n* = 5, 7.1%) cancers. The primary focus of LB biomarkers among the included publications was ctDNA (*n* = 56, 75.6%), followed by CTCs (*n* = 29, 41.4%), and cfDNA (*n* = 19, 27.1%).

**Table 1:** Summary of key characteristics of included publications

Classification	Papers (*N* = 75), n (%)^a^
**Country of Corresponding Author**	
China	17 (24)
United States	15 (20)
Australia	5 (7)
Italy	4 (6)
Canada	4 (6)
Other	30 (37)
**Publication Type and Study Method**	
Narrative Review	63 (84)
Empirical Study	6 (8)
Non-empirical *Perspective*	3 (4)
Clinical Study	3 (4)
**Cancer Type**	
All Cancer (All Stages)	23 (31)
Lung / NSCLC	9 (11)
Colorectal cancer	7 (9)
Head and Neck / Oral	5 (7)
Gastric	4 (6)
Bladder	3 (4)
Breast	3 (4)
Other^a^ *(Prostate, Renal, Lymphoma, Glioblastoma, Melanoma, Ovarian)*	17 (33)
**Liquid Biopsy Focus**^**a**^	
ctDNA	56 (76)
CTC	29 (41)
cfDNA	19 (27)
Broad (*including a combination of ctDNA, CTC, cfDNA*)	12 (17)

^a^Column may not equal 74 due to missing values and overlap in some categories

*Abbreviations*: *cfDNA* cell free DNA, *ctDNA* circulating tumour, *CTCs* circulating tumour cells, *NSCLC* Non-small cell lung cancer

The majority of the 75 included articles were narrative reviews (*n* = 63, 84%). There were six exemplar non-narrative review articles, which included four empirical studies [4, 49–51] and two non-empirical studies [52, 53]. In addition to five clinical studies [54–59] The narrative reviews explored various aspects of LB in cancer care, including but not limited to implementation challenges. Some reviews were not tumour specific, describing the range of potential uses for LB across the patient journey, while others were cancer and treatment specific, for example focusing on potential uses of LB for immunotherapy in small cell lung cancer [60]. Many reviews were focused on blood specimens [61] while others explored other specific sample types including urine, and a subset focused on early detection [13, 62].

Of the exemplar articles, one empirical study was a retrospective clinical study that investigated the real-world adoption of LB for colorectal cancer, and was conducted by Fischer et al. (2022) [51]. The study found that research evidence was not being applied in clinical practice and recommended clinical trials to be developed to assist with clinical guideline development. The second empirical study, by Woof et al. (2022), was a qualitative investigation assessing the perceptions of early-stage melanoma patients about the reporting of LB results used to routinely monitor for recurrence [49]. A third study reported an Australian workshop by Ijzerman et al. (2021), where 70 local experts met in 2020 to gather their perspectives on the challenges and opportunities of LB across various cancer types [4]. Attendees agreed that early detection was the least developed use case for LB, with higher levels of evidence supporting the application of LB for monitoring of minimal residual disease and disease progression surveillance [4]. Finally, the Consensus Statement by Dasari et al. (2020) was a unique article representing the work of a committee appointed by the US National Cancer Institute to examine ctDNA use cases and integration into clinical care for colorectal and rectal cancers [50]. The statement produced six broad recommendation areas including, addressing barriers to integration of ctDNA into CRC care, assay characteristics, management of minimal residual disease, management of rectal cancer, monitoring of metastatic disease and tracking clonal dynamics. These categories were further developed in order to advance LB by standardizing pre-analytic and analytic issues and identifying how to proceed in a selection of use-cases [50]. The two non-empirical studies were both position papers, by Rolfo et al. (2020) and Russo et al. (2021) respectively. They explored the challenges and opportunities of cfDNA in clinical practice and the need to standardize both pre-analytic procedures and analytic methods in the molecular profiling of solid tumours [52, 53]. The clinical studies explored various aspects of implementation, detection and personalised treatment strategies. The study by Shick et al. (2022) identified health professionals views on ctDNA as a transformative tool for cancer management and tumour dynamics [54]. The study Peng et al. (2020) evaluated current practices in reporting next generation sequencing results for ctDNA, highlighting key challenges and opportunities to advance precision oncology utilising LB [55]. The clinical studies by Henriksen et al. (2024) evaluate the effectiveness of ctDNA testing in colorectal cancer in a Danish cohort aimed at improving early detection, similarly, Burns et al. (2023) looked at barriers faced when testing for NSCLC in urban hospital settings [56, 57]. Finally the two studies by Kramer et al. (2023) both evaluated the economic impact of implementing ctDNA testing in the Netherlands and future scenarios for integrating circulating tumour DNA testing into oncology, identifying key facilitators and barriers for clinical adoption [58, 59].

### Keyword analysis

**Fig. 4:**
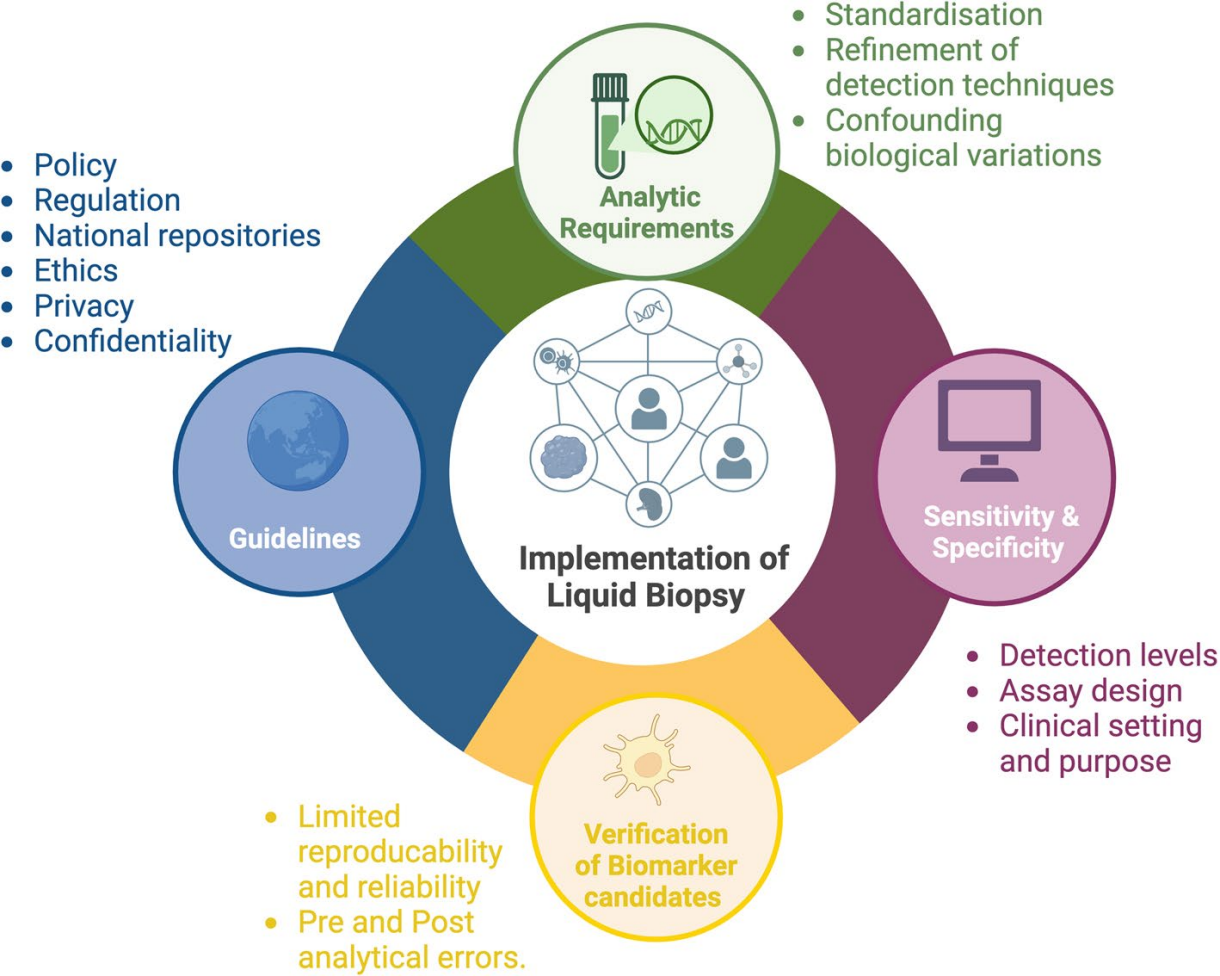
Network of co-occurring keywords appearing in more than one publication. Each circle (node) is a keyword, and each line (edge) indicates co-occurrence. The size of each node corresponds to the frequency of keyword use, and colours represent different topic areas as described in the key

Figure 4 demonstrates the network analysis of author-supplied keywords, highlighting key topic areas interlinked across multiple articles. A significant number of studies focused on ctDNA, cfDNA or CTCs, with specific attention to next-generation sequencing, potential biomarker applications, or cancer-specific disease focus. Broadly applying these approaches could enhance our understanding of the multi-dimensional nature and variability of LB, potentially improving implementation strategies across various clinical settings.

### Factors influencing liquid biopsy implementation

**Fig. 5:**
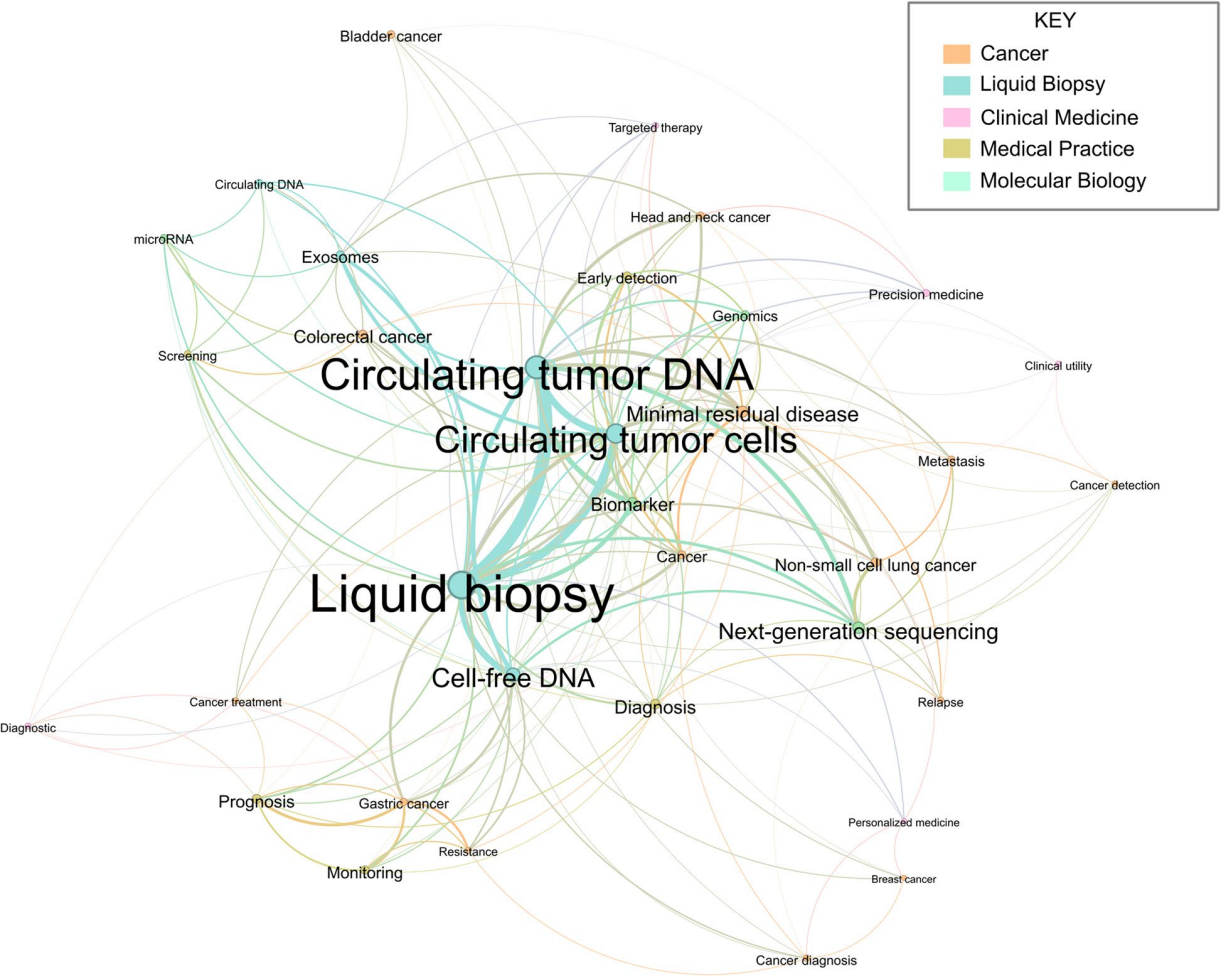
Summary of the key factors influencing the implementation of liquid Biopsy identified in the included articles. The figure highlights the key factors influencing the implementation and effectiveness of liquid biopsy technologies, emphasizing areas where improvements can facilitate their broader application in clinical practice

From the 70 articles included in this study, we identified four main and strongly inter-related conceptual categories and their associated barriers and facilitators to the implementation of LB in cancer care: (1) pre-analytic and analytic requirements; (2) sensitivity and specificity; (3) verification of biomarker candidates; and (4) guidelines. A summary of these categories is presented in Fig. 5.

#### Pre-analytic and analytic requirements

Several articles highlighted barriers related to the need for standardization and lack of optimisation of pre-analytical procedures, from sample collection through pre-processing, which can significantly impact the sensitivity of cfDNA analysis [63–65] (Table 2). These barriers include challenges with plasma volume, transit time, time intervals between blood collection and plasma isolation, centrifugation methods, purification techniques, and temperature control [52, 62, 66, 67]. For instance, Warton et al. (2017) compared standard EDTA-containing tubes with specialized tubes that contained preservative solutions like Streck cfDNA BCT and Qiagen PAXgene Blood cfDNA. These specialised tubes were found to assist in the prevention of haemolysis and reduce cfDNA fragment degradation, and were thus a facilitator to blood collection in clinical settings by extending the time available before processing [68].

**Table 2:** Pre-analytical barriers identified and recommendations for optimisation

Barriers	Current Challenges	Recommendations for Optimisation
Confounding biological and environmental variations	Influence of pre-sampling factors such as diet, medication use, age and gender on quality/quantity of isolated analytes. Sampling factors including collection time, blood draw technique, sample volume and collection processes	Need for standardized sample collection protocols and guidelines to improve rigor and reproducibility Following reporting guidelines for cfDNA and specific biomarkers should be implemented [77]
Storage and handling	Inconsistencies due to decay rates, stability of analytes, temperature sensitivity, cell lysis/haemolysis and application differences among samples. Differential effects of collection tubes (e.g. EDTA blood vs Streck tubes) [78]	Define optimal storage conditions (time and temperature) and sample storage volume to allow for more accurate results in molecular diagnostics [3]
Isolation of LB analytes (CTC, ctDNA, cfDNA	Lack of standardized protocols for processing analytes	Need to establish standard processing protocols [79]
Establishing population norms	Poor understanding of normal biological variation in individualized biofluids	Creation of known standards to better account for biological variation in biofluids and technical variation [3]

*Abbreviations: cfDNA* cell free DNA, *ctDNA* circulating tumour, *CTCs* circulating tumour cells, *LB* Liquid Biopsy, *EDTA* Ethylenediaminetetraacetic acid

The absence of standardized protocols in the pre-analytical phase remains a considerable barrier to LB implementation [69]. A key method of overcoming this barrier is local investment in the human and laboratory infrastructure that is required to integrate assays effectively into the clinical workflow [18]. Notably, the standardization of the analysis of CTCs, extracellular vesicles, and other LB modalities is lacking behind ctDNA due to the availability of ctDNA detection and analysis technologies [49, 70]. Nevertheless, institutions planning to use commercial ctDNA profiling assays (e.g., Oncomine cell-free nucleic acid assays, AVENIO ctDNA targeted kit, QIAseq targeted DNA panels) must conduct on-site validation prior to using the assay as a companion diagnostic tool in a clinical trials or routine practice [71].

Articles discussed the complex challenges associated with clinical workflow, particularly for local pathology laboratories that may struggle to match the competencies of central laboratories. While central laboratories may benefit from economies of scale, making them likely to dominate larger multigene ctDNA assays, decentralised testing could be possible for single-gene or small multigene (fewer than five genes) assays; however, this has the potential to reduce testing sensitivity [72–75].

Education and training of hospital personnel were frequently identified as crucial facilitators for successful implementation. Even in settings where assays are conducted off-site, trained staff are required for blood draws, initial sample processing, running and maintaining quality control of assays, and interpreting assay reports [76]. Facilitating this through workplace seminars or incorporating it into university curricula would enhance the effective application of these assays in clinical practice [3].

#### Factors affecting sensitivity & specificity of analysis

A key barrier is the low sensitivity of LB in certain circumstances, particularly when there are lower levels of biomarkers in plasma, such as in early-stage cancer. Higher disease burden solid tumours are more likely to shed tumour-derived DNA into the bloodstream, which facilitates the detection of cancer via LB [14]. The sensitivity and specificity of LB analyses are crucial considerations, with distinct implications depending on the clinical setting. For example, in the adjuvant setting, sensitivity is the most critical factor for detecting MRD after initial treatment, as it ensures that even low levels of remaining cancer are identified. In the metastatic setting, where cancer volume is higher, high specificity ensures that a positive test accurately detects disease and minimizes the risk of false positives that could lead to inappropriate treatment decisions [6, 63]. The balance between sensitivity and specificity is crucial depending on the treatment decision, often outweighing considerations of disease stage. Treatment escalation demands high specificity to avoid unnecessary interventions, while de-escalation requires high sensitivity to ensure MRD is detected and treatment is not withheld from those who need it.

ctDNA levels vary depending on cancer type and cancer site. For instance, intracranial tumours are less likely to release detectable levels of DNA into the bloodstream compared to extracranial tumours due to the blood–brain barrier limiting the cfDNA release into circulation [65]. In such cases, cerebrospinal fluid (CSF) can serve as an alternative and valuable source for detecting tumour-derived DNA, offering a more direct route to access ctDNA from intracranial lesions, however obtaining CSF samples is invasive hence LB is often preferred for monitoring resistance and identifying molecular targets. Utilizing CSF may improve diagnostic sensitivity and provide critical insights into disease progression where blood-based LB is less effective [65, 80]. Research into alternative LB biofluids such as urine, breast milk, bile and saliva shows promise due to their unique biomarker profiles, but significant barriers remain [3]. Urine is easily accessible but prone to dilution and variability in biomarker concentration or analyte levels [81]. Breast milk offers insights into breast tissue dynamics but is limited to lactating individuals and presents ethical challenges, including potential misunderstandings about its use for diagnostic or research purposes, potential privacy concerns regarding genetic and health data, and the need for strict confidentiality to prevent misuse or discrimination [15]. Bile contains valuable biomarkers for hepatobiliary cancers but requires invasive sampling methods [28], similar to intracranial lesions. Saliva, which is easy to collect, transport and store is one of the best candidates for the advancement of point-of-care medicine, where individuals are able to easily monitor their health status by using portable convenient tools such as smartphones [82, 83]. While blood plasma is the most studied medium for ctDNA analysis, non-blood fluids such as urine, saliva, breast milk, and bile are gaining attention for their potential applications in various cancer detection.

Common challenges across all biofluids include a lack of standardization in collection and processing, technical limitations in detecting low-abundance biomarkers, and insufficient validation across diverse populations.

Non-shedders refer to cancer patients whose tumours release little to no detectable ctDNA into the bloodstream or other biofluids, posing a significant challenge for LB applications in cancer diagnosis, prognosis, and treatment monitoring [91]. Research suggests that non-shedding may result from various biological factors including; tumour type and location, tumour biology and size, vascularisation, and immune response [14, 53, 92]. Non-shedding is particularly common in cases where tumour size is small, in the early stages of disease, or in the case of low-grade and indolent cancers, which produce less ctDNA due to limited tumour burden or reduced necrosis rates. Host factors, including individual metabolic variations and clearance mechanisms, further influence ctDNA levels [55].

The variability in non-shedding samples is strongly dependent on tumour type and stage [55, 93]. For example, ctDNA levels have been reported in over 75% of patients with advanced pancreatic, ovarian, colorectal, bladder, gastroesophageal, breast, melanoma, hepatocellular, and head and neck cancers [94]. However, this percentage drops below 50% for primary brain, renal, prostate, or thyroid cancers, with even lower levels observed in patients with localized tumours [3]. Analytical limitations also contribute to false-negative results in non-shedders, necessitating tissue biopsy in some cases [63].

MRD detection in non-shedders faces similar challenges, as ctDNA levels may fall below detection thresholds despite persistent microscopic disease. Non-DNA biomarkers, including extracellular vesicles, and circulating tumour cells (CTCs), offer complementary diagnostic tools [95]. Advances in next-generation sequencing (NGS) and digital PCR (dPCR) improve sensitivity, enabling the detection of extremely low ctDNA levels, aiding in non-shedder cases [96]. Combined modalities, such as integrating LB with imaging techniques like PET-CT, further enhance diagnostic accuracy by correlating ctDNA shedding with tumour activity and burden [97]. Despite these advancements, significant barriers remain. Deeper mechanistic insights into ctDNA shedding variability, longitudinal studies to clarify its relationship with disease progression, and broader exploration of novel biomarkers are critical for improving LB utility in non-shedders.

#### Need for verification of biomarker candidates

The articles included in this review identified various barriers and facilitators associated with the implementation of biomarker-based LBs in cancer care. Despite the generation of potential biomarkers for clinical diagnostics, fewer than 1% are reported to successfully transition into clinical practice [98]. This low success rate can be attributed to several barriers encountered throughout different phases of biomarker discovery and clinical application. Key barriers include limited reproducibility of published findings, use of custom in-house assays/technologies, lack of standardised pre-analytical and analytical conditions that hinder the reliable use and implementation of biomarkers in LB [99].

Many of these barriers are concentrated in the pre-analytical phase and relate to lack of standardisation of collection and transport and laboratory procedures [75, 100, 101]. Other issues likely relate to differences in the cancers under study and the clinical spectrum included in the study. The lack of reproducibility in biomarker studies complicates the pathway from discovery to clinical application, as inconsistent results hinder the validation and approval of biomarkers for clinical use [102]. Facilitators that emerged from the literature include advancements in standardising pre-analytical and analytical procedures, which can assist in mitigating errors and improve reproducibility [70, 74]. Additionally, increasing the use of robust validation studies and developing more sensitive assays are crucial for overcoming these barriers [74, 99, 103].

#### Guidelines

Several barriers and facilitators were related to policy and regulation associated with the implementation of LB. A major barrier was the current lack of clear and comprehensive guidelines, which impacts the accuracy, reliability, and consistency of LB tests across laboratories [54, 73, 104]. The absence of standardised regulations contributes to variability in test results, posing risks to patient safety and the overall credibility of LB as a diagnostic tool [104]. Conversely, facilitators include initiatives by consortia, such as the European Liquid Biopsy Society (ELBS) and BLOODPAC (https://www.bloodpac.org/), which are working to standardize pre-analytical and analytical methods [32, 105]. These initiatives are addressing variability and enhancing test reliability, thereby aiding progress and facilitating the integration of LB into clinical practice [3].

A further barrier involved the ethical challenges associated with LB testing. Issues such as the risk of overdiagnosis and the psychological impact of detecting genetic mutations with uncertain clinical relevance were highlighted as significant concerns [80]. This situation is particularly concerning in early-stage cancers, where the potential for harm from unnecessary treatments is elevated. Patients receiving results that indicate genetic mutations without clear guidance on their implications often experience anxiety, stress and confusion. This emotional burden of such diagnoses, especially in the context of cancers with variable survival rates, can undermine the potential benefits of early detection and monitoring [106]. Therefore, it is essential to establish comprehensive guidelines that not only ensure the accuracy and reliability of LB tests but also the ethical and psychosocial aspects of patient care. These concerns emphasise the need to not only focus on technical accuracy but also address the broader implications of LB on patient care [107]. Examples of this have been demonstrated in the DYNAMIC study, which was the first prospective, randomized study to demonstrate that ctDNA could guide therapeutic decisions in patients with stage II colorectal cancer [34]. Similarly, The BFAST trial demonstrated the clinical utility of blood-based NGS as a method to inform clinical decision-making in ALK-positive NSCLC [108].

Finally, high costs associated with LB tests and the potential lack of insurance coverage are a barrier to widespread adoption. The cost of LB ranges from $500USD to $3,000USD per test, depending on the complexity of the assay and the healthcare system. In comparison, imaging modalities such as CT scans and MRI scans can cost between $300 and $5,000 with PET scans generally costing between $2,000 and $5,000 [109]. Tissue biopsies can range between $1,000 and $2,500 and cost is influenced by the type of biopsy and subsequent analysis [110, 111]. These costs can vary based on factors such as technology, location, and insurance coverage. The variance in costs often limits patient access and contributes to disparities in care [14]. Moreover, cost-effectiveness calculation must consider gains in life years and quality-adjusted life years (QALYs), savings on overtreatment and hospitalisation [112].

## Discussion

The results of this scoping review illustrate the complex interplay of factors that are likely to influence the successful implementation of LB in clinical practice. Our results highlight an overwhelming number of barriers to LB implementation, most prominently at the pre-analytical stage. Early detection and screening are currently viewed as the gold standards and key facilitators for integrating LB into routine cancer care. Unlike traditional invasive procedures such as colonoscopies, mammograms and tissue biopsies [113, 114], LB offers less invasive alternatives for cancers such as brain and NSCLC cancer, which traditionally depend on more invasive techniques like cerebrospinal fluid and tissue biopsies [29, 115]. However, to improve the prospects of LB implementation in clinical practice, attention must focus on overcoming the identified barriers in the pre-analytical stage. These challenges include stringent laboratory and personnel requirements, which demand specialized training and resources often lacking in current settings [54]. Disease specificity further complicates the situation, as the effectiveness of LB varies depending on the type of cancer, requiring tailored approaches that are not universally applicable [32]. Additionally, the reliance on biomarker-based LB introduces variability in accuracy and sensitivity, raising concerns about the reliability of results [116, 117]. Policy and regulation constraints, which are still evolving, also create hurdles by limiting the widespread adoption and standardization of LB techniques. Collectively, these factors negatively impact the clinical implementation of LB, hindering its potential to improve patient outcomes [4, 7].

The recent increase in empirical papers and structured reviews indicates that LB as a field and scientific technique may be maturing and formalising as a research paradigm. This trend suggests aspirations to further develop theory and capture more knowledge to strengthen the empirical evidence base. Our investigation identified a relative lack of experimental studies compared to narrative research. This aligns with the recognition of the difficulty in translating LB into clinical research and some perspectives that LB may not yet be ready for implementation. Qualitative approaches have predominated in the empirical literature, reflecting a focus on gaining an in-depth understanding of LB, rather than solely conducting experimental studies. The few experimental studies that discussed implementation explored health professionals’ views on ctDNA in hereditary cancer management and reporting practices on next-generation sequencing for tumour DNA analysis [54]. We also identified two position papers, and one workshop focused on LB implementation in cancer management [52, 53]. Key issues raised by these studies underscore the need to address pre-analytical variables impacting standardization. ctDNA leads in clinical implementation among LB techniques, but clinical utility remains the main hurdle. The challenges for ctDNA differ from those of CTCs, extracellular vesicles and CTC, which use distinct assay principles and produce varied results.

The integration of LB in cancer care is promising, but the lack of large-scale studies, standardized cohorts, and coordinated committees limits its widespread implementation [107, 111]. From an implementation science perspective, this study contributes to standardizing LB protocols, which is crucial for developing clinical practice guidelines (CPG). Establishing a unified approach can lead to more consistent and reliable patient outcomes, ultimately improving patient satisfaction and the overall healthcare system [70]. Standardization can not only enhance the accuracy and reliability of LB but also accelerates its integration into routine clinical practice, ensuring that patients receive effective and personalized care.

There are currently five LB diagnostic assays that have been approved for clinical use by the US Food and Drug Administration (FDA; Table 3). There clinical use ranges from treatment selection in breast cancer, non–small-cell lung cancer, prostate cancer, colorectal cancer as well as diagnostic for all solid tumours [118] (Table 4).

**Table 3:** Overview of ctDNA Derived from Urine, Saliva, Breast Milk, and Bile for Cancer Detection

Body Fluid	Source of ctDNA	Cancer Types	Challenges
Urine [81, 84–86]	Direct shedding from tumours in the urinary tract	Urothelial cancers (bladder, kidney, upper urinary tract)	Lower ctDNA concentrations compared to plasma
		Non-urinary tract cancers (lung, colorectal, breast)	Requires highly sensitive detection techniques (e.g. dPCR, NGS) Urine DNA degradation varies with storage time and temperature
Saliva [87, 88]	Local shedding from tumours in head and neck	Head and neck cancers (HPV/EBV-associated, nasopharyngeal)	Requires complementary techniques to enhance sensitivity, address tumour heterogeneity and overcome low concentrations
	Systemic circulation via blood-saliva barriers	Other cancers (lung and pancreatic)	Risk of contamination from oral microbiota or epithelial cells
Breast Milk [64, 89]	Direct shedding from breast tissue, including malignant cells	Breast cancer (e.g. BRCA1/2 and TP53 mutations)	Limited to lactating women and low ctDNA fraction in breast milk
		Rarely systemic cancers	Sparse research data and protocol standardization
Bile [90, 91]	Tumour cell shedding from biliary tract, liver, pancreas or gallbladder	Hepatobiliary cancers (cholangiocarcinoma, gallbladder)	Invasive sampling methods (e.g. ERCP)
		Pancreatic cancer (e.g. KRAS mutations) and liver metastases	Difficulty in standardization of pre-analytical factors

*Abbreviations*: *BRCA* breast cancer gene 1/ 2, *ctDNA* circulating tumour, *dPCR* digital polymerase chain reaction, *EBV* Epstein-Barr Virus, *ERCP* Endoscopic retrograde cholangiopancreatography, *HPV* Human papillomavirus, *KRAS* Kirsten rat sarcoma, *NGS* Next-generation sequencing

**Table 4:** FDA-approved LB cancer assays

Year	Cancer Type	Biomarker/Target	LB Test Name	Clinical Use
2016	Non-Small Cell Lung Cancer	EGFR mutation	Cobas® EGFR Mutation Test v2	Detection of EGFR mutations for targeted therapy (erlotinib, osimertinib) in NSCLC [119]
2017	Breast Cancer	PIK3CA mutation	Therascreen® PIK3CA RGQ PCR Kit	Detection of PIK3CA mutations for the use of alpelisib in breast cancer [120]
2020	Multiple Cancers	Tumour mutational burden (TMB)	FoundationOne® Liquid CDx	Comprehensive genomic profiling and assessment of tumour mutational burden [121]
2020	Multiple Cancers	Multiple gene alterations	Guardant360® CDx	Genomic profiling to guide targeted therapies across multiple cancer types [118]
2021	Colorectal Cancer	KRAS mutation	Guardant360® CDx	Detection of KRAS mutations for anti-EGFR therapy exclusion [118]
2022	Breast Cancer	ESR1 mutations	Guardant360® CDx	Detection of ESR1 mutations to guide endocrine therapy decisions [118]
2022	NSCLC	KRAS	Agilent Resolution ctDx assay (resolution Bioscience Inc)	Detection of KRAS gene [122]

*Abbreviations*: *CDx* Companion diagnostic, *EGR* Estimated glomerular filtration rate, *ESR1* Estrogen receptor protein1, *KRAS* Kirsten rat sarcoma virus, *PIK3CA* phosphatidylinositol-4,5-bisphosphate 3-kinase catalytic subunit alpha, *NSCLC* Non-small cell lung cancer

One key step towards clinical implementation is the standardization of critical pre-analytical variables [123]. This challenging process requires careful workflow design and validation through large comparability schemes [66]. Several organizations and committees worldwide are currently working toward LB implementation in clinical practice, covering various aspects of this multifaceted procedure. Notably, the International LB Standardization Alliance (ILSA) is working toward the global application of LB in oncology practice [124]. In Europe, the European LB Society (ELBS), as an extension to the CANCER-ID project, is currently developing guidelines to foster LB research and industry interactions (www.elbs.eu) [125]. In the US, BloodPAC addresses gaps where guidelines are lacking or inadequate [126]. The International Society of LB (ISLB) (https://islb.info/) provides recommendations and guidelines for designing and validating LB assays for successful clinical implementation [52]. As LB technologies continue to evolve, collaborating with key stakeholders at all stages is crucial.

LB show great promise in oncology for detecting early relapse or resistance through ctDNA, potentially enabling clinicians to adjust treatments before clinical symptoms appear. Ongoing trials, like TRACERx in lung cancer and DYNAMIC-III in colorectal cancer, are exploring whether treatment adjustments based on ctDNA levels can improve outcomes [127, 128]. However, several challenges remain, including determining the appropriate thresholds for intervention, variability in ctDNA shedding across cancer types, and the timing of interventions. While LBs have been useful in guiding therapy changes in cancers with known resistance mutations, such as EGFR-driven lung cancer, strong evidence that these adjustments lead to better survival rates is still emerging. Prospective clinical trials are crucial to establish whether LB-guided interventions lead to improved clinical outcomes. Randomized studies are needed to compare LB-based adjustments to standard care, and adaptive trials may offer a way to personalize treatment based on real-time ctDNA dynamics. For instance, serial ctDNA monitoring for anti-EGFR resistance mutations identified colorectal cancer patients who benefited from rechallenge with anti-EGFR therapy, with one-third of patients lacking detectable circulating resistance variants showing further responses upon rechallenge [129]. Similarly, in the APPLE Phase II randomised trial, the detection of EGFR inhibitor resistance variants in non-small cell lung cancer patients receiving EGFR inhibitors allowed for earlier transitions and responses to the third-generation EGFR inhibitor, Osimertinib [130]. However, standardization in ctDNA interpretation, cost-effectiveness analysis, and further evidence from randomized trials are necessary to determine the true impact of LB-guided therapies on long-term survival and disease management.

### Future implications and guidelines

The future of LB holds immense potential for transforming cancer diagnostics and treatment. While this review emphasizes the importance of incorporating multiple analytes, such as cfDNA, CTCs, and circulating extracellular vesicles, into LB assays, ctDNA remains a more established and proven marker, with significant standardization challenges still ahead for the others. Future research should focus on identifying which cancer types can benefit from LB-based assays, considering factors such as known etiology, type, and the extent of sample cfDNA or ctDNA, along with the underlying disease and treatment resistance mechanisms [88, 131].

There is a pressing need to establish consensuses on integrating emerging technologies into existing workflows to enhance sampling and improve detection levels at the laboratory stage. A significant barrier to the clinical implementation of LB is the lack of large prospective longitudinal cohorts to validate these technologies [3]. Leveraging the work of various stakeholders in the LB field: epidemiologists, health economists, policymakers and incorporating their views to address the multiple pre-analytical variables is critical to the standardization of reported LB modalities [7]. The six exemplars highlighted in the review have preliminary demonstrated the potential advancements that can be made to advance LB. For instance, working groups of various stakeholders focusing on specific tumour streams, in line with the National Cancer Institute (NIC)—Colorectal Cancer Group should be formed for other cancer types [132]. Additionally, it is essential to understand clinician and patient perspectives on LB, as their acceptance and trust in these technologies will influence their adoption in clinical practice [49]. Gaining insights into their concerns and expectations can help guide the development and implementation of these assays. Moreover, there is a need for innovative prospective studies that can evaluate the role of LB in achieving a shift in cancer care, reducing unnecessary invasive procedures and workups, improving detection efficiency, and providing comparative information alongside the current standard of care [93]. The ultimate objective of LB technologies should be to develop cost-effective, robust diagnostic tests and longitudinal monitoring tools that complement existing cancer care methods.

### Strengths and limitations

To our knowledge, this is the first review to specifically identify barriers and facilitators related to the implementation of LB in cancer care. A key strength of this review is the inclusion of a broad range of articles, encompassing various publication types and study methods, which collectively provide insights into the challenges and opportunities for implementing LB in clinical practice. Additionally, we conducted a novel keyword analysis, as well as inductive thematic coding to identify key topic areas and relationships between articles. This approach has helped to highlight the critical issues currently facing the field, with the aim of directing attention to areas in need of further research.

However, the results of this review should be interpreted in the context of several limitations. As a scoping review, we did not include a grey literature search, which may have broadened our understanding of the field and uncovered additional barriers and facilitators that were not discussed. Additionally, while we focused on articles specifically addressing the clinical implementation of LB, many were included in our final analysis if they contained sections that alluded to these issues. Furthermore, articles were included if they used the terms CTC, ctDNA or cfDNA, even if the keyword ‘LB’ was not explicitly mentioned. Additionally, the potential for LB to outperform other diagnostic methods in the metastatic setting compared to other clinical contexts was seldom discussed explicitly in the reviewed literature. While some papers discussed this characteristic, it was not consistently reported. A few studies emphasised the enhanced utility of LB in metastatic contexts, particularly for guiding treatment decisions and monitoring therapeutic response, but this was not a routine focus of the review [63, 111]. Finally, we acknowledge that exosomes are a component of LB with potential clinical utility, however, articles solely reporting on exosomes were excluded from our review.

### The significance of various LB techniques

ctDNA and CTCs are the most widely studied biomarkers inLB, each offering distinct advantages and limitations in cancer detection, monitoring, and treatment. CtDNA is highly sensitive for detecting genetic mutations, tumour heterogeneity and minimal residual disease particularly in advanced cancers [3]. Serial ctDNA monitoring allows for early disease detection, non-invasive tracking of treatment response and identification of actionable mutations. However, the sensitivity of ctDNA is influenced by the tumour’s shedding capacity, meaning non-shedding tumours or those with low tumour burden may result in false negatives [63].

In contrast, CTCs provide a direct cellular representation of the tumour, capturing not only genetic information but also phenotypic traits like epithelial-to-mesenchymal transition, which is a hallmark of metastasis and therapy resistance [88, 92]. CTCs are valuable for monitoring metastatic disease and studying tumour biology, but they are less sensitive than ctDNA, particularly in early-stage cancers [88]. Detection of CTCs is also technically challenging, as it relies on specific markers, which may not capture all tumour cell types. Furthermore, the clinical utility of CTCs is still under validation with standardized detection methods lacking.

When comparing ctDNA and CTCs, ctDNA generally offers superior sensitivity for early detection, particularly in cancers with high DNA shedding [133]. CTCs, are particularly useful for phenotypic analysis of the tumour cells. While other biomarkers, including vesicle-derived nucleic acids such as those from exosomes and microvesicles show promise, their clinical application is still in the research phase and they are not yet suitable for direct comparison with ctDNA or CTCs due to limited data [4]. It should also be noted that there is emerging evidence for contrasting and combining CTC and ctDNA which could hold great future potential [134]. Ultimately, the combination of ctDNA and CTCs may enhance sensitivity and provides complementary prognostic and predictive information.

## Conclusion

The widespread implementation of LB testing in routine clinical practice hinges on two critical factors: (a) the lack of standardization and (b) the need for a clear demonstration of the clinical utility of specific assays. Progress is being made through the concerted efforts of various organizations working towards fully integrating LB tests into clinical practice. The evidence discussed in this review contributes to the ongoing dialogue surrounding the growth, development, and evolving nature of the LB field. While the field is becoming more established, the practical application of LB remains limited at present. Focusing on and addressing the barriers identified in this review will significantly enhance the clinical utility of LB for treatment selection. Overcoming these challenges in the coming years is expected to lead to a profound change in the practice of cancer care, by introducing LB as a pivotal tool for the real-time assessment and management of tumour evolution.

## Supplementary Information

Supplementary Material 1. Appendix 1. Database Search Strategy.

Supplementary Material 2. Appendix 2. Coding Structure and Definitions for keyword analysis.

Supplementary Material 3. Appendix 3. Articles included in the study (*n* = 75).

## Data Availability

The datasets used and/or analysed during the current study are available from the corresponding author on reasonable request.

## Abbreviations

cfDNA: Cell-free DNA
CPG: Clinical Practice Guideline
CTC: Circulating Tumour Cells
ctDNA: Circulating Tumour DNA
ELBS: European LB Society
ILSA: International LB Standardization Alliance
ISLB: International Society of LB
LB: Liquid Biopsy
MeSH: Medical Subject Headings
MRD: Minimal Residual Disease
NIC: National Cancer Institute
NSCLC: Non-small cell lung cancer
PRISMA-ScR: Preferred Reporting Items for Systematic Reviews and Meta-Analyses extension for Scoping Reviews
