# Supplementary material for: A scoping review of factors influencing the implementation of liquid biopsy for cancer care
Source: J Exp Clin Cancer Res. 2025 Feb 12;44:50. doi: 10.1186/s13046-025-03322-w (PMC11817833; doi:10.1186/s13046-025-03322-w)
Supplement: Supplementary file 1 — Supplementary Material 1. Appendix 1. Database Search Strategy. [file 13046_2025_3322_MOESM1_ESM.docx]

# Liquid biopsy search strategy

## Databases to search:

Embase, Scopus, PubMed & Web of Science

Total articles from search: 4837

- Embase – 1624
- PubMed – 770
- Scopus – 1134
- Web of Science – 1309

Total articles after deduplication:

- 2158 references kept
- 2679 references removed

### Embase – 1624

### <https://simsrad.net.ocs.mq.edu.au/login?url=http://ovidsp.ovid.com/ovidweb.cgi?T=JS&NEWS=N&PAGE=main&SHAREDSEARCHID=3Xx7triQLzRawGteIG6CTCqzMqZO485t7mE0cBpNAxDaLQxjU35OZMcjxpVF8dD19>

1. 1 and 2 and 3 and 4


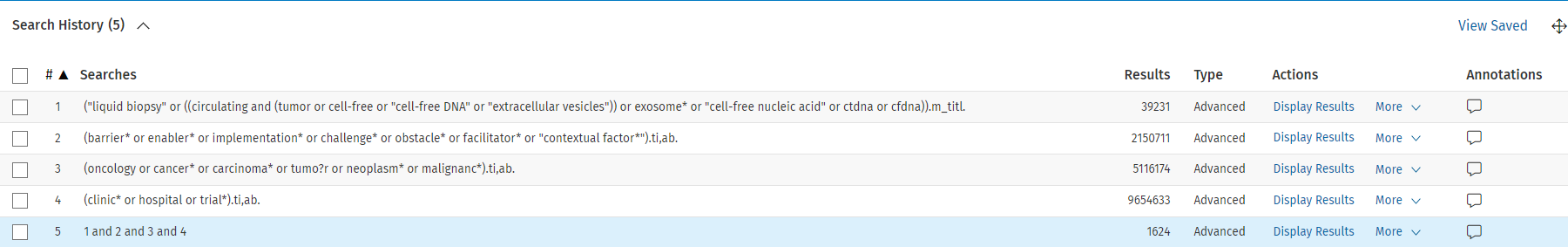


### Scopus – 1134

"liquid biopsy" OR ( ( circulating ) AND ( tumor OR cell-free OR "cell-free DNA" OR "extracellular vesicles" ) OR exosome* OR "cell-free nucleic acid" OR ctdna OR cfdna )

AND

barrier* OR enabler* OR implementation* OR challenge* OR obstacle* OR facilitator* OR "contextual factor*"

AND

oncology OR cancer* OR carcinoma* OR tumo?r OR neoplasm* OR malignanc*

AND

clinic* OR hospital OR trial*


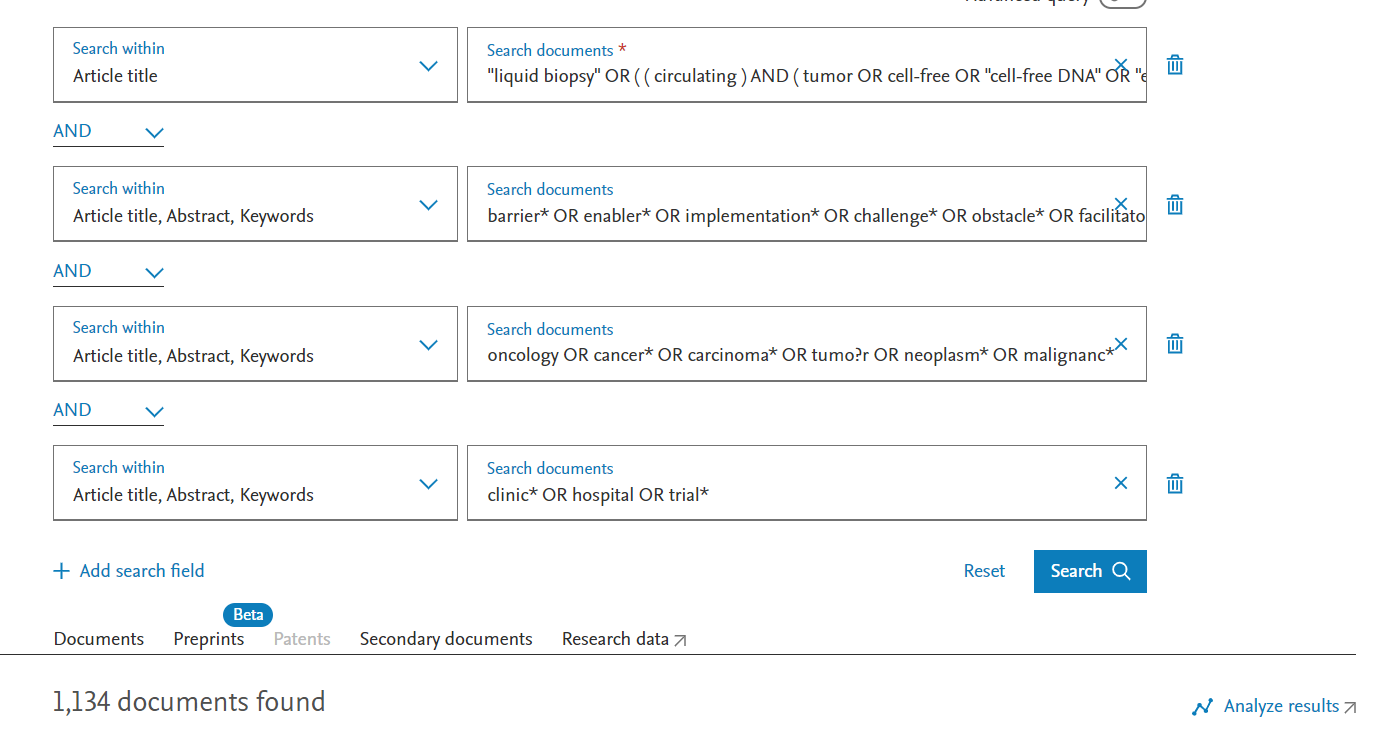


### PubMed – 770 results

(((liquid biopsy[Title] OR circulating tumor DNA[Title] OR (circulating tumo?r DNA[Title] OR ctDNA[Title] OR cfdna[Title] OR circulating cell-free tumo?r DNA[Title] OR circulating cell-free DNA[Title] OR circulating tumo?r cell*[Title] OR circulating extracellular vesicles[Title] OR exosome*[Title] OR cell-free nucleic acid [Title])) AND (barrier*[Title/Abstract] OR enabler*[Title/Abstract] OR challenge*[Title/Abstract] OR facilitator*[Title/Abstract] OR implementation[Title/Abstract] OR obstacle*[Title/Abstract] OR contextual factor*[Title/Abstract])) AND (cancer*[Title/Abstract] OR oncology[Title/Abstract] OR carcinoma*[Title/Abstract] OR tumo?r*[Title/Abstract] OR neoplasm*[Title/Abstract] OR malignanc*[Title/Abstract])) AND (clinic*[Title/Abstract] OR hospital[Title/Abstract] OR trial*[Title/Abstract])

### Web of Science -1309

<https://www.webofscience.com/wos/alldb/summary/8f4a226c-b6f5-4c32-bc7c-f5fad4cf8031-cf5400c1/relevance/1>

"liquid biopsy" OR ( ( circulating ) AND ( tumor OR cell-free OR "cell-free DNA" OR "extracellular vesicles" ) OR exosome* OR "cell-free nucleic acid" OR ctdna OR cfdna )

AND

barrier* OR enabler* OR implementation* OR challenge* OR obstacle* OR facilitator* OR "contextual factor*"

AND

oncology OR cancer* OR carcinoma* OR tumo?r OR neoplasm* OR malignanc*

AND

clinic* OR hospital OR trial*
